# Long-term survival analysis of cementless large-diameter head metal-on-metal total hip arthroplasty

**DOI:** 10.1007/s00402-022-04633-9

**Published:** 2022-10-05

**Authors:** Elli Holappa, Jukka Kettunen, Hannu Miettinen, Heikki Kröger, Simo Miettinen

**Affiliations:** 1 Department of Orthopaedics, Traumatology and Hand Surgery, Kuopio University Hospital, PO Box 1777, 70211 Kuopio, Finland; 2 Faculty of Health Sciences, University of Eastern Finland, Yliopistonranta 1, 70210 Kuopio, Finland

**Keywords:** Total hip arthroplasty, Metal-on-metal, Survival, Complications, Revision

## Abstract

**Introduction:**

The aim of this retrospective study was to study the long-term survival and reasons for revisions of a single-type, large-diameter head (LDH) metal-on-metal (MoM) implant. A special study interest was to find the threshold level for revision in terms of adverse reaction to metal debris (ARMD).

**Materials and Methods:**

In this cohort study, we retrospectively reviewed 234 patients (253 hips) who received 38 mm head size LDH MoM total hip arthroplasties (THAs) between 01 January 2004 and 31 December 2009 at our institution. Patient symptoms, conventional radiographs, magnetic resonance imaging (MRI) findings and whole blood metal ions were studied.

**Results:**

The median follow-up time was 11.5 years. The Kaplan–Meier cumulative survival estimate of the implant was 89.6% at 10 years and 82.9% at 14.6 years. Overall, 34/253 (13.4%) hips were revised during the follow-up period and of these; 19/34 (55.9%) were revised due to ARMD. The prevalence of ARMD was 12.4% (29/234 patients).

**Conclusions:**

The survival of the implant was on a moderate level as the 10-year cumulative survival rate was 89.6%. The exact threshold level for ARMD revision cannot be determined due to multiple variables affecting factors in re-operation decision-making.

## Introduction

Total hip arthroplasty (THA) is the treatment of choice for hip disability [1]. In the late 1990s, metal-on-metal (MoM) implants with large-diameter heads (LDHs) were introduced [2]. A LDH MoM (36 mm or larger) has shown to increase implant stability and decrease dislocation risk, but beneficial effects large head size implant are negated beyond a size of 38 mm due to increased volumetric metal wear [3]. After a promising start, several studies with these second-generation MoM bearings, including LDHs, have shown an increased risk to adverse reactions to metal debris (ARMD) and higher-than-expected revision rates [4–6]. ARMD is an umbrella term describing joint failures associated with pain, sterile periprosthetic effusions, necrosis and metallosis [7]. The reason for ARMD is that MoM bearing surface and trunnion interface produces nanometer-sized metal particles and this volumetric wear further causes histological changes in periprosthetic tissue, like necrosis and inflammatory reaction [8–10].

Due to potential ARMD, the usage of second-generation MoM implants decreased and in 2010 Medicines and Healthcare products Regulatory Agency of the United Kingdom (MHRA) published a safety alert for all MoM hip replacements [11]. Subsequently, several authorities have published guidelines for systematic follow-up [12–15]. Several cut-off values have been used, which has led to different, country-specific follow-up protocols [16]. Criticism towards the variation of different country-specific guidelines has been aimed as a threshold level for LDH MoM implant revision due to ARMD also variates [17].

Arthroplasty register studies include only limited data of a specific head size 38 mm MoM bearing THA implants and highly detailed survivorship studies of these implants are very scarce. The primary outcome of this study was to determine long-term survival of this single type implant M2a-38™/Bi-Metric LDH MoM THA (Biomet, Inc., Warsaw, Indiana, USA). The secondary outcomes were to evaluate adverse events and reasons for revisions. Consequently, the reasons and definitions for ARMD are well known, but the threshold level for revision of the current implant due to ARMD remains somewhat unclear [17]. A specific study interest was to find out if there was an unambiguous threshold level for the revision due to ARMD in terms of patient-reported outcomes, radiological analysis and whole blood metal ion levels when national guidelines and international definitions of ARMD where utilised [11–19].

## Materials and methods

### Study information and patient population

A total of 233 patients (253 hips) underwent a primary LDH MoM THA between 01 January 2004 and 31 December 2009 at the Kuopio University Hospital (KUH) and all consecutive patients were included in this retrospective cohort study. Patients had routine clinical follow-up with conventional radiographs at 3 months postoperatively. Hereafter follow-up was implemented according to national guidelines which included a clinical follow-up, conventional radiograph and whole blood Co and Cr levels in every other year [12]. Magnetic resonance imaging (MRI) was performed in those cases where patient had any symptoms or blood Cr or Co level was above 5 μg/L [12]. According to national guidelines, revision due to ARMD was considered when (1) very high blood metal ion levels (above 20 μg/L) were found regardless of symptoms or MRI findings, (2) there were osteolysis, clear pseudotumor of other signs of ARMD in the MRI or (3) patient had pain or other symptoms related to THA regardless of the blood metal levels or MRI findings [12]. The end of follow-up was set to either 31 December 2019, upon death of the patient or date of revision due to any reason. During the follow-up period, 50/233 (21.5%) of enrolled patients died and did not experience revision operation.

Patient characteristics (age, gender, body mass index, ASA classification [physical status classification by American Society of Anaesthesiologists]), operative data, Harris Hip score (HHS), whole blood chromium (Cr) and cobalt (Co) ion levels, adverse events and any symptoms related to THA were harvested from the medical registry of KUH. HHS was systematically collected from all the patients in every follow-up visit and only fully completed questionnaires were included to this study. Acetabular component inclination and anteversion angles were measured from the postoperative radiographs. Special interests were in cases where acetabulum component inclination was > 50° and where component position was out of the Lewinnek safe zone (5°–25° anteversion and 30°–50° inclination) [20]. Metal artifact reduction sequence magnetic resonance imaging (MARS-MRI) was used to evaluate possible ARMD. The studied implant system was the 38 mm head size MoM bearing with cementless stem (M2a-38™/Biomet, Inc., Warsaw, Indiana, USA).

### Outcome measures

The primary outcome measure was the long-term survival of the implant. The secondary aims were to determine adverse events (major and minor) and the reasons for revision [ARMD, loosening of the implant, periprosthetic joint infection (PJI), malposition of the implant, dislocation, abductor muscle damage with pain or periprosthetic fracture]. A major adverse event was defined as a revision due to any reason, symptomatic or non-symptomatic ARMD without revision, PJI, and periprosthetic fracture and dislocation. Minor adverse events included distracting leg length difference or hip pain prompting the patient to contact the hospital/orthopaedist outside of normal control visits.

### Adverse reaction to metal debris

In the current study, ARMD was defined as follows, according to previous studies [18, 19]:

1. diagnosis was made in revision based on presence of periprosthetic pseudotumour,
2. presence of a pseudotumour, found in MARS-MRI (or computed tomography [CT]) scan or
3. Co or Cr level in whole blood was ≥ 10 μg/L.

MARS-MRI findings were classified by a radiologist, based on the Hart et al. method of classification of pseudotumours [21]. Patients were classified into three groups according to Cr and Co ion levels, based on the Finnish guideline of MoM patient follow-up [12] and definition of definite ARMD in previous studies [18, 19]. The groups in this study were (1) < 5 μg/L (no ARMD), (2) 5–10 μg/L (probable ARMD) and (3) over 10 μg/L (definite ARMD).

### Statistical analyses

Statistical analyses were carried out using SPSS Version 27.0.0 (IBM SPSS Inc., Chicago, IL, USA). The normality distribution of the data was checked and the Mann–Whitney *U* test was used for non-parametric data, Chi-square analysis and Fisher–Freeman–Halton exact test were used for categorical data and an independent samples *T*-test was used to compare continuous data between groups. Kaplan–Meier analysis was used to study the implant survival and revision due to ARMD and in addition, competing-risks analysis was performed where death was set as a competing outcome factor for revision. Cox regression analysis was used to evaluate the most common risk factors for revision due to ARMD according to the literature [6, 7, 22]. Risk factors were age, gender, positions of the components, metal ions and Hart classification of pseudotumours. All *p*-values < 0.05 were considered statistically significant.

## Results

### Patients and demographics

A total of 233 patients (253 hips) were evaluated, of which 20/233 (8.6%) had similar implants bilaterally. The median follow-up time was 11.5 years (range, 3 days–15.8 years). Patients’ baseline characteristics are displayed in Table 1.

**Table 1:** Patient demographic characteristic. Non-revised patients are compared to revised patients

	All hips (*n* = 253)	Non-revised (* n* = 219)	Revised (* n* = 34)	*p*-value
	*n* (%)	*n* (%)	*n* (%)	
*Gender*				0.38^b^
Female	109 (43.1)	92 (42.0)	17 (50)	
Male	144 (56.9)	127 (58.0)	17 (50)	
*ASA-classification*				0.68^e^
1	9 (3.6)	8 (3.7)	1 (2.9)	
2	56 (22.1)	43 (19.6)	13 (38.2)	
3	78 (30.8)	67 (30.6)	11 (32.4)	
4	12 (4.7)	10 (4.6)	2 (5.9)	
Unknown	98 (38.7)	91 (41.6)	7 (20.6)	
*Operation indication*				0.48^e^
Primary arthrosis	197 (77.9)	173 (79.0)	24 (70.6)	
Hip dysplasia	7 (2.8)	6 (2.7)	1 (2.9)	
Rheumatoid arthitis	7 (2.8)	5 (2.3)	2 (5.9)	
Hip fracture	17 (6.7)	15 (6.8)	2 (5.9)	
Avascular necrosis	17 (6.7)	13 (5.9)	4 (11.8)	
Other	8 (3.2)	7 (3.2)	1 (2.9)	
*Operation side*				0.17^b^
Right	121 (47.8)	101 (46.1)	20 (58.8)	
Left	132 (52.2)	118 (53.9)	14 (41.2)	
*Surgical approach*				0.52^e^
Lateral	5 (2.0)	4 (1.8)	1 (2.9)	
Posterolateral	248 (98.0)	215 (98.2)	33 (97.1)	
*Acetabulum component position*				
Inclination angle, median° (range)	46.0 (20–72)	46.0 (20–68)	47.5 (26–72)	0.49^c^
Anteversion angle, median° (range)	24.0 (3–51)	24.0 (3–44)	32.0 (4–51)	0.05^c^
*Acetabular component position at Lewinnek safe zone*^a^				0.15^b^
Yes	103 (40.7)	93 (42.5)	10 (29.4)	
No	150 (59.3)	126 (57.5)	24 (70.6)	
*Inclination angle > 50°*				0.29^b^
Yes	77 (30.4)	64 (29.2)	13 (38.2)	
No	176 (69.6)	155 (70.8)	21 (61.8)	
*Age, mean (SD, range)*	62.8 (8.3, 33–87)	63.1 (8.4, 33–87)	60.7 (7.6, 43–78)	0.11^d^
*BMI, median (range)*	27.8 (18.0–40.0)	27.9 (19.5–40.0)	27.5 (18.0–36.5)	0.54^d^
*Size (mm) of the acetabulum component, median (range)*	53.4 (44–62)	53.5 (44–62)	52.9 (48–62)	0.30^d^

*BMI* body mass index, *SD* standard deviation, *ASA* the physical status classification by American society of anaesthesiologists, *mm* millimeters

^a^Lewinnek safe zone (5°–25° anteversion and 30°–50° inclination) described by Lewinnek et al. [20]

^b^Chi squared test

^c^Mann–Whitney *U* test

^d^Independent samples *t*-test

^e^Fisher–Freeman–Halton exact test

### Survival analysis

**Fig. 1:**
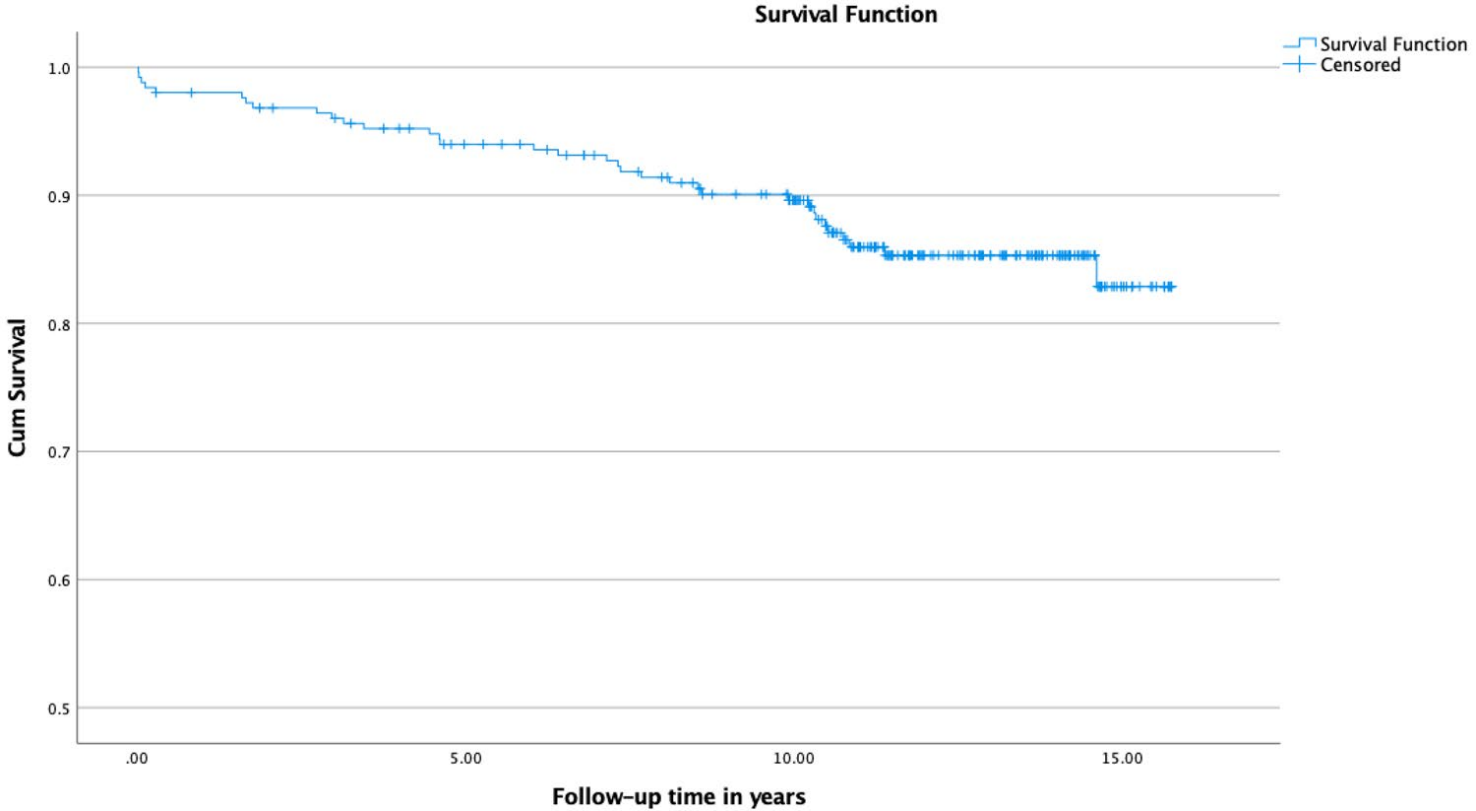
Kaplan–Meier survival analysis of time to revision due to any reason. The estimates for the cumulative survival were 94.0% at 5 years, 89.6% at 10 years and 82.9% at 14.6 years (SE 0.238, CI 95% 13.8–14.8)

**Fig. 2:**
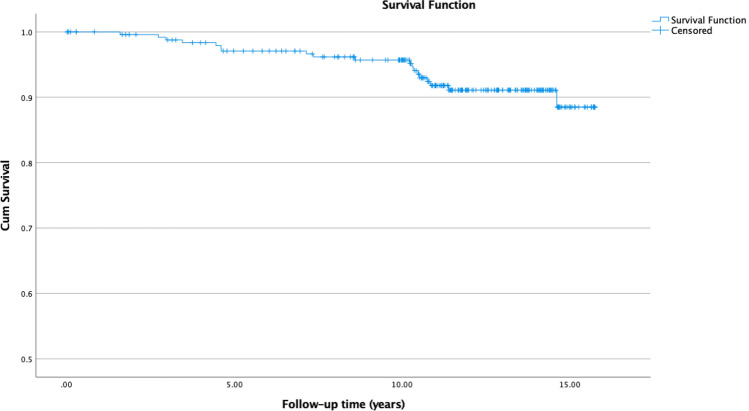
Kaplan–Meier survival analysis of time to revision due to ARMD. The estimates for the cumulative survival were 97.1% at 5 years, 95.2% at 10 years and 88.5% at 14.6 years (SE 0.167, CI 95% 14.7–15.4)

The cumulative Kaplan–Meier survival estimate for no need for revision due to any reason was 94.0% at five years, 89.6% at 10 years and 82.9% at 14.6 years (Standard error [SE] 0.2, 95% CI 13.8–14.8) (Fig. 1). For no need for ARMD revision, it was 97.1% at five years, 95.2% at 10 years and 88.5% at 14.6 years (SE 0.2, 95% CI 14.7–15.4) (Fig. 2).

### Adverse events and risk factors for revision

In total, 34/253 (13.4%) of hips were revised. The details of adverse events and revisions are provided in Table 2. The median time to revision due to any reason was 6.8 years (range, 3 days–14.6 years). The number of hips with at least one major adverse event was 52/253 (20.6%). Consequently, 18/253 (7.1%) of hips had major adverse events, but no revision. Three patients experienced very early intraoperative complications, which led to revision within one month for the following reasons: component malposition (revised after three days), PJI (seven days) and loosening of acetabulum component (21 days). In addition, three-fourths of the revisions due to acetabulum component loosening were performed within two years after primary operation (on day 21, in 1.7 years and in 1.6 years) and all these patients were female.

**Table 2:** Adverse events and revision surgeries after total hip arthroplasty

	All hips (* n* = 253)
*Adverse event*	*n* (%)
No	176 (69.6)
Yes	77 (30.4)
Major	52 (20.6)
ARMD	29 (11.5)
Loosening of acetabulum component	4 (1.6)
PJI	5 (2.0)
Abductor muscle damage and pain	2 (0.8)
Component malposition	3 (1.2)
Dislocation	3 (1.2)
Periprosthetic fracture	5 (2.0)
Loosening of femur component	1 (0.4)
Minor	25 (9.9)
Contacted an orthopaedist because of distracting chronic groin/hip pain	9 (3.6)
Contacted hospital because of intensive pain lasting a couple of days or weeks	11 (4.3)
Distracting leg-length difference	5 (2.0)
*Revision*	
No	219 (86.6)
Yes	34 (13.4)
Reason for revision	
ARMD	19 (7.5)
Loosening of acetabulum component	4 (1.6)
PJI	3 (1.2)
Abductor muscle damage and pain	2 (0.8)
Component malposition	3 (1.2)
Dislocation	1 (0.4)
Periprosthetic fracture	1 (0.4)
Loosening of femur component	1 (0.4)

*ARMD* adverse reaction to metal depris, *PJI* periprosthetic joint infection

Altogether, 29/253 (11.5%) of hips had definite ARMD and 19/29 (65.5%) of them underwent revision. The median time to revision due to ARMD was 8.6 years (range, 1.6–14.6 years). Ten patients with definitive ARMD did not experience revision, of which seven were asymptomatic, two had mild symptoms (hip pain) and one had symptomatic ARMD with revision indication; however, the operation was not performed during follow-up (patient was diagnosed in 12/2019). Among bilateral MoM implant patients, 4/20 (20.0%) were diagnosed with AMRD, of which one experienced revision due to ARMD and three did not experience revision.

Competing-risks analysis showed that age (HR 0.95, 95% CI 0.92–0.98, *p* = 0.003) was a risk factor for revision and other studied factors [female gender (HR 1.34, 95% CI 0.69–2.60, *p* = 0.38), bilateral vs. unilateral LDH MoM THA (HR 0.52, 95% CI 0.15–1.78, *p* = 0.30), acetabulum component outside the safe zone (HR 0.68, 95% CI 0.33–1.40, *p* = 0.29) and acetabulum component inclination > 50° (HR 1.32, 95% CI 0.68–2.56, *p* = 0.42)] were not risk factors.

### Patient-reported outcomes

The details of clinical outcomes are listed in Table 3. Altogether, 31/34 (91.2%) of the revised hips were painful, chronically or occasionally, before revision. The HHS was collected before revision or at the latest clinical follow-up and it was available for 169/219 (77%) of the non-revised patients and for the revised patients it was available for only for 15/34 (44%) of the patients. The median HHS of the non-revised patients was 96.0 (range, 15–100) and for revised patients it was 93.0 (range, 69–100) (*p* = 0.31).

**Table 3:** Clinical outcomes of the monitored hips

	Non-revised (* n* = 219)	Revised (* n* = 34)	*p*-value
	*n* (%)	*n* (%)	
*Symptoms*			
No symptoms	136 (62.1)	–	
Pain in hip area, chronic	26 (11.9)	24 (70.6)	
Pain in hip area, occasional	49 (22.4)	7 (20.6)	
No pain, but other symptoms (sounds, clicking, loose feeling, cramps, numbness)	8 (3.7)	3 (8.8)	
*ARMD*^a^	10 (4.6)	19 (55.9)	< 0.01^c^
A, B, symptoms	0	6 (17.6)	
A, symptoms	3 (1.4)	4 (11.8)	
A, no symptoms	6 (2.7)	–	
B, symptoms	–	6 (17.6)	
B, no symptoms	1 (0.5)	–	
C, symptoms	–	3 (8.8)	
*Conventional radiographs*			< 0.01^e^
Monitored hips	219 (100)	34 (100)	
No findings in latest screening	198 (90.4)	11 (32.4)	
Osteolysis around acetabulum component	2 (0.9)	6 (17.6)	
Osteolysis around femur component	5 (2.3)	2 (5.9)	
Osteolysis around both components	1 (0.5)	1 (2.9)	
Periproshtetic fracture	4 (1.8)	4 (11.8)	
Excessive inclination angle of acetabulum component	3 (1.4)	5 (14.7)	
Definite loosening of acetabular component	–	2 (5.9)	
Partly loosening of acetabular or femur component	5 (2.3)	–	
Other (thick cortex, osteolysis combined with fracture or loosening, malposition of components)	1 (0.5)	3 (8.8)	
*MARS-MRI*			0.34^e^
Monitored hips	29 (13.2)	18 (52.9)	
No findings	16 (7.3)	5 (14.7)	
Pseudotumor	9 (4.1)	10 (29.4)	
Hart 1	3 (1.4)	2 (5.9)	
Hart 2a	2 (0.9)	2 (5.9)	
Hart 2b	2 (0.9)	5 (14.7)	
Hart 3	2 (0.9)	1 (2.9)	
Pseudotumor, no other findings	6 (2.7)	6 (17.6)	
Pseudotumor combined with abductor muscle damage or osteolysis	3 (1.4)	4 (11.8)	
Abductor damage only	3 (1.4)	3 (8.8)	
Osteolysis only	1 (0.5)	-	
Metal ion levels in whole blood			
Number of measurements	155 (70.8)	19 (55.9)	
Bilateral metal-on-metal implant (any brand) at time of Co/Cr measurements	45 (20.5)	7 (20.6)	
Division into three groups^b^			< 0.01^e^
Co or Cr < 5 µ/L	114 (73.5)	6 (31.6)	
Co or Cr ≥ 5 and ≤ 10 µ/L	38 (24.5)	2 (10.5)	
Co or Cr > 10 µ/L	3 (1.9)	11 (57.9)	
Median Co level, µ/L			
All (range)	3.3 (0.3–13.0)	22.0 (0.7–193.0)	< 0.01^d^
Bilateral, any brand (range)	2.7 (0.4–8.5)	57.1 (1.3–193.0)	0.03^d^
Unilateral (range)	3.3 (0.3–13.0)	20.5 (0.7–69.0)	< 0.01^d^
Median Cr level, µ/L			
All (range)	2.1 (0.5–7.9)	13.0 (0.7–57.9)	< 0.01^d^
Bilateral, any brand (range)	2.5 (0.6–4.2)	17.6 (1.0–57.9)	0.18^d^
Unilateral (range)	2.0 (0.5–7.9)	11.3 (0.7–39.5)	< 0.01^d^

*ARMD* adverse reaction to metal depris, *HHS* Harris hip score, *MARS*-*MRI* metal artifact reduction sequence magnetic resonance imaging, *Co* cobalt, *Cr* chromium

^a^A: pseudotumor found in MARS MRI, B: Co or Cr level in whole blood over 10 µ/L, C: diagnosed in revision surgery

^b^No ARMD (Co or Cr < 5 µ/L), probable ARMD (Co or Cr ≥ 5 and ≤ 10 µ/L), Definitive ARMD (Co or Cr > 10 µ/L)

^c^Chi squared test

^d^Mann–Whitney *U* test

^e^Fisher–Freeman–Halton exact test

### Radiological analysis

Conventional radiographs were available for all hips at least on the first control visit after primary operation. In total, 47/253 (18.6%) of hips underwent MARS-MRI during follow-up. Pseudotumours were found in 19/47 (40.4%) of screened hips. Among screened and revised hips, 10 had pseudotumours; four did not have pseudotumours, but were considered as ARMD revisions, and four had neither pseudotumours nor ARMD. The median time between MARS-MRI and revision operation was, on average, 3.4 months (range, 1.8–12.7 months). The incidence of pseudotumours was 19/253 (7.5%), while 6/19 (31.6%) of the pseudotumours were symptomless. Asymptomatic pseudotumours were classified as Hart 3 (one case), 2b (one case), 2a (two cases) and 1 (two cases). Altogether, 29/47 (61.7%) of scanned hips were not revised, of which 16 hips had symptoms and 13 hips were symptomless. The median time between the primary operation and the latest MARS-MRI among non-revised hips was 8.3 years (range, 3.0–14.6 years).

### Metal ion analysis

During follow-up, 174/233 (74.7%) of patients underwent metal ion analyses, of which 52/174 (29.9%) had bilateral MoM implants of any brand at the time of analysis and 40/174 (23.0%) of patients had the implant of this study bilaterally. Between the bilateral (any brand) and unilateral MoM groups, there were statistical differences in metal ion values with the highest values appearing in the bilateral group (Table 3).

In 19/34 (55.9%) of the revision cases, metal analyses were taken before revision, and ARMD was the main reason for revision in 15/19 (78.9%) of these cases. The median metal ion concentrations before ARMD revision were 51.4 µ/L for Co (range, 1.3–193.0 µ/L) and 21.1 µ/L for Cr (range, 1.0–57.9 µ/L). In 11/19 (57.9%) cases, analyses were performed after revision, with median values of 2.5 µ/L for Co (range 1.4–6.5 µ/L) and 3.2 µ/L for Cr (range 0.6–6.7 µ/L). On average, Co and Cr ion levels after ARMD revision decreased 92.8% and 87.5%, respectively.

### Cox regression analysis

The Cox regression model showed that age (hazard ratio [HR] 0.98, 95% confidence interval [CI], 0.94–1.02, *p* = 0.27), female gender (HR 1.30, 95% CI 0.66–2.54, *p* = 0.45), acetabulum component anteversion (HR 1.03, 95% CI 1.00–1.06, *p* = 0.09), acetabulum component inclination (HR 1.04, 95% CI, 0.99–1.08, *p* = 0.09), acetabulum component size (HR 0.95, 95% CI 0.86–1.05, *p* = 0.33) and pseudotumour size on MRI (Hart 1: HR 1.47, 95% CI, 0.31–6.97, *p* = 0.63; Hart 2a: HR 1.41, 95% CI 0.30–6.73, *p* = 0.67; Hart 2b: HR 2.62, 95% CI 0.84–8.16, *p* = 0.10; Hart 3: HR 0.90, 95% CI 0.11–7.23, *p* = 0.92) were not risk factors for revision due to ARMD. However, Co level (HR 19.10, 95% CI 8.25–44.22, *p* < 0.001) and Cr level (HR 27.50, 95% CI 10.40–72.50, *p* < 0.001) were found to be risk factors for ARMD revision.

## Discussion

This study investigated the survival rate of the 38 mm LDH MOM implant (M2a-38™/Biomet) and found that 34/253 (13.4%) hips had undergone revision within median follow-up of 11.5 years. The cumulative survival rate was 94.0% at five years, 89.6% at 10 years and 82.9% at 14.6 years. These study results are similar to those of previous studies in which the cumulative survival rate varied from 95 to 97% at five years, 85 98% at 10 years and 74% at 13 years [23–25].

Patient characteristics did not show any other statistical significance risk factor for ARMD revision than age. Reasons other than ARMD for revisions (osteolysis around acetabulum component, periprosthetic fractures and component malposition) were on acceptable levels when compared to other similar studies [23, 25]. ARMD caused 19/34 (55.9%) of all revisions, as 7.5% (19/253) of all hips were revised due to ARMD, and overall, 11.5% (29/253) of hips had definite ARMD. According to the National Joint Registry of the United Kingdom (NJR) 17th report, the most common reasons for revision among THAs are aseptic loosening, dislocation and ARMD, while MoM bearings have the highest incidence of ARMD [26]. In previous studies with LDH MoM bearings, 31–69% of all revisions were performed due to ARMD [18, 25] and the prevalence of definite ARMD has been 11–14% within a mean follow-up period of 3.8–6.7 years [18, 19, 27]. This study found there is a notable increase in the number of ARMD revisions at 10 years after the primary operation. However, according to competing-risks factor analysis, higher age of the patient seems to protect from ARMD revision. One apparent reason for this finding might be that some high-age patients, with inherent indication for ARMD revision, were so fragile that the general risks of surgery outweigh more than the potential benefits of revision, and thus, revision was not performed.

In this study, Co or Cr level in whole blood ≥ 10 μg/L was considered definite ARMD. This study showed that high Cr and Co levels most likely led to ARMD revision as the Cr and Co levels of revised patients were statistically significantly higher compared to non-revised patients. Revised patients with any type of bilateral MoM THA had higher metal ion levels in general than unilateral 38 mm LDH MOM patients. In addition, the Cox regression model showed that only high Cr and Co levels were risk factors for ARMD revision and other studied factors (age, gender, acetabulum component position and pseudotumour on MRI) were not statistically significant risk factors, which support previous study findings [6, 27].

A pseudotumour was found in 19/47 (40.4%) of screened hips and of these pseudotumours, 6/19 (31.6%) were symptomless. Previous studies reported 7–61% incidence of asymptomatic pseudotumours among MoM hips [21, 28–30]. All ten hips that were revised due to a pseudotumour experienced symptoms, at least chronic hip pain. Three symptomatic pseudotumours were not revised. One of them (Hart 3) had occasional moderate pain and loose feeling/clicking, one (Hart 2b) had occasional moderate pain and the third (Hart 1) had chronic pain. Presumably, none of these symptoms were distracting enough to indicate revision. Altogether, 13/19 (68.4%) of identified pseudotumours and 34/47 (72.3%) of MARS-MRI-screened hips were symptomatic.

Osteolysis on conventional radiograph was found in 17/253 (6.7%) of hips, of which nine (52.9%) were revised. One cohort with MRI-imaged asymptomatic LDH MoM THAs showed a 7% prevalence of osteolysis [28]. The implant model in our study previously showed median ion values in whole blood after implantation of 2.6–4.8 µ/L for Co and 1.0–2.5 µ/L for Cr [23, 31]. In our study, the results of the ion levels were higher. Some authorities recommend using a cut-off level of 7 µ/L [14, 15], yet it has been shown to have high specificity, but low sensitivity, in terms of detecting implant failure [32]. The incidence of groin pain with LDH MoM implant is reported to be 17–36% [30, 33, 34]. In the current study, a total of 50/253 (19.8%) patients (24 before revision and 26 non-revised) experienced chronic groin pain.

The strength of this study is its detailed demographic and radiologic analyses. To our knowledge, this study has currently the largest sample size and longest follow-up period with this particular implant. The limitations of this study include its retrospective nature, which could have been minimised by a prospective study design. In addition, there was lack of HHS scores and radiographs right before revision. The number of pseudotumours may be underestimated due to non-systematic MARS-MRI scanning. There were possible cases of loss of follow-up as some patients did not attend appointments or the follow-up protocol (clinical examination, Cr/Co measurements, conventional radiographs) within the preferred every two-year schedule was not fulfilled.

In conclusion, the overall long-term survival of the 38 mm LDH MoM implant was at a moderate level with a 10-year survival rate of 89.6%. This study found an 11% ARMD rate, in which revision numbers remained quite stable during the first ten years of follow-up and alarmingly increased after that time period. The threshold level for ARMD revision was considered high as only 66% of the diagnosed ARMD patients was eventually revised and follow-up still continues with the rest. Based on these results, hip symptoms, osteolysis on conventional radiograph, pseudotumour on MRI and elevated blood metal ions all most likely lead to ARMD revision. In general, the exact threshold level for ARMD revision cannot be established due to multiple variables affecting factors in re-operation decision-making. Instead, patient-dependent factors should be taken into consideration as an entirety and follow-up should be continued regular especially when 10 years of follow-up has been passed with the currently studied implant.

## Data Availability

Not applicable.
